# Health care seeking behaviour for children with acute childhood illnesses and its relating factors in sub-Saharan Africa: evidence from 24 countries

**DOI:** 10.1186/s41182-021-00385-1

**Published:** 2021-12-14

**Authors:** Sanni Yaya, Emmanuel Kolawole Odusina, Nicholas Kofi Adjei

**Affiliations:** 1 School of International Development and Global Studies, University of Ottawa, Ottawa, ON Canada; 2 The George Institute for Global Health, Imperial College London, London, UK; 3 Department of Demography and Social Statistics, Faculty of Social Sciences, Federal University, Oye, Ekiti Nigeria; 4 Department of Public Health, Policy and Systems, University of Liverpool, Liverpool, L69 3BX UK

**Keywords:** Health care seeking behaviour, Childhood illness, Under-five children, Mothers, Global health, Sub-Saharan Africa

## Abstract

**Background:**

Childhood illnesses and mortality rates have declined over the past years in sub-Saharan African countries; however, under-five mortality is still high in the region. This study investigated the magnitude and factors associated with health care seeking behaviour for children with childhood illnesses in 24 sub-Saharan African countries.

**Methods:**

We used secondary data from Demographic and Health Surveys (DHSs) conducted between 2013 and 2018 across the 24 sub-Saharan African countries. Binary logistic regression models were applied to identify the factors associated with health care seeking behaviour for children with acute childhood illnesses. The results were presented using adjusted odds ratios (aOR) with 95% confidence intervals (CIs).

**Results:**

Overall, 45% of children under-5 years with acute childhood illnesses utilized health care facilities. The factors associated with health care seeking behaviour for children with acute illnesses were sex of child, number of living children, education, work status, wealth index, exposure to media and distance to a health facility.

**Conclusions:**

Over half of mothers did not seek appropriate health care for under-five childhood illnesses. Effective health policy interventions are needed to enhance health care seeking behaviour of mothers for childhood illnesses in sub-Saharan African countries.

**Supplementary Information:**

The online version contains supplementary material available at 10.1186/s41182-021-00385-1.

## Background

Childhood illnesses such as fever, diarrhea and acute respiratory infection (ARI) are serious child health issues in low income countries [1, 2]. Globally, in 2015, about 5.9 million under five children died due to preventable causes [1, 3–5], and approximately three out of every four under-five deaths occur due to ARI, diarrhea and fever [1, 6]. This phenomenon is more pronounced in low-income and middle-income countries [7], where childhood deaths and illnesses are serious health issues [1, 8], especially in sub-Saharan Africa [4, 9–11]. Although under-five mortality has generally declined globally, it is still a critical issue in sub-Saharan Africa [12, 13], as the region accounts for about 50% of under-five preventable mortality [14].

Prior evidence showed that pneumonia and diarrhea are among the most common causes of childhood illnesses and deaths in many African countries [15, 16]. Addressing the burden of childhood illnesses is  critical in sub-Saharan Africa,  because  sub-Saharan African countries have a greater burden of childhood illnesses in contemporary time [17–19]. In addition, progress to achieve Sustainable Development Goal (SDG) 3.2 (elimination of preventable child death, reduction in neonatal mortality to less than 12 per 1000 live births and those of under-five mortality to less than 25 per 1000 live births for every country by 2030) is slower, and without the necessary policies and interventions, sub-Saharan Africa may not meet the SDG 3.2 target by 2030 [20]. There is evidence that at least one child dies out of every 12 births in sub-Saharan Africa before age five compared to one out of every 147 in developed countries [3]. The majority of under-five mortality in sub-Saharan Africa is due to infectious and parasitic diseases, such as malaria, respiratory infections, meningitis and diarrhea [5, 13, 14, 21]. Diarrhea, for instance, is a major cause of morbidity and mortality globally, as it accounts for 1.3 million deaths among children under-5 years of age each year [21]. Although childhood illnesses can be managed efficiently in sub-Saharan Africa, evidence suggest poor health care seeking behaviour among mothers for childhood illnesses in the region [18, 22]. Evidence based information is thus needed on health care seeking behaviour of mothers for effective policies and interventions in sub-Saharan Africa [22]. Meanwhile, prompt and adequate health care seeking behaviour interventions among mothers can substantially reduce childhood mortality due to childhood illnesses in low-income and middle-income countries [10].

Efforts have been made globally and by various countries in sub-Saharan Africa to reduce morbidity and mortality of under-five resulting from childhood illnesses through policies that will promote child health care services among mothers [23–26]. To achieve the Sustainable Development Goal target of at most 25 deaths per 1000 live births by the year 2030, there are efforts and high-level commitments towards addressing the issues. For instance, at the global level are the Integrated Management of Childhood Illness (IMCI) strategy developed by the World Health Organization (WHO), the Every Woman Every Child Strategy and the Partnership for Maternal Newborn and Child Health [3, 10, 27, 28]. Moreover, many countries in sub-Saharan Africa have policies and programmes that are aimed at reducing under-five morbidity and mortality. For instance, Nigeria introduced the Maternal and Child Health Policy in 1994, National Immunization Policy and Standards Practice in 1996 and Breastfeeding Policy in 1999 [14]. Many African countries have also embraced the International Child Rights Policy, emphasizing and promoting child health care services among [29].

Although prior studies have provided some evidence on factors affecting childhood illnesses in low-income and middle-income countries [30–32], there is little knowledge on health care seeking behaviour among mothers for childhood illnesses in sub-Saharan Africa. The factors associated with health care seeking behaviour from studies conducted in different countries include knowledge of danger signs, occupation, residence, education, age, marital status, birth order, mass media exposure [1, 13, 33] and income [34]. Community level promotion of prompt health care seeking behaviour among mothers for childhood illnesses to enhance the health care of under-five children has been emphasised [5, 35]. Meanwhile, under-five mortality is still high in sub-Saharan Africa despite the progress made in the last few decades. Proper health care seeking behaviour of mothers for childhood illnesses can prevent or reduce the magnitude of child mortality resulting from childhood illnesses [10, 36, 37]. This study, therefore, seeks to examine the factors associated with under-five children illnesses such as diarrhea and fever and health care service utilization in sub-Saharan African countries. Assessing health care services for childhood diseases and associated risk factors may help prevent and reduce under-five morbidity and mortality in sub-Saharan African countries [5].

## Methods

The study used secondary data from the Demographic and Health Surveys (DHSs) of 24 countries in sub-Saharan Africa. The countries were selected if the surveys were conducted between 2010 and 2018 [33, 38], and outcome and explanatory variables were available. The DHSs use multi-stage stratified sampling method [33, 38, 39]. These DHSs are nationally representative and comparable surveys conducted worldwide in more than 85 countries [40]. The surveys usually collect a wide range of self-reported and objective data with a strong focus on indicators of reproductive health, fertility, child and maternal health, nutrition, mortality, and self-reported health behaviours among adults [41]. The sample for the final analyses was 98,590. It included children who had diarrhea and/or fever or cough in the 2 weeks preceding the surveys, whether they sought private or public health care or not. The country specific details are presented in Additional file 1.

### Measurement of variables

#### Outcome variable

The outcome of interest was under-five children with incidence of diarrhea and/or cough or fever in the past 2 weeks before the surveys. Those who went for consultation in a public or private health care service provider and those who did not go were classified as users and non-users, respectively [14].

#### Explanatory variables

The explanatory variables considered were maternal education (no formal education, primary and secondary plus), age of mother (15–24, 25–34 and 35–49), occupation of mother (working and not working), wealth index (poorest, poorer, middle, richer and richest), marital status (never married and ever married), residence (urban and rural), distance to health facility (experience no difficulty in getting to a health facility/not a problem in getting to a health facility or experienced difficulty in getting to a health facility/a problem to in getting to a health facility), media access (no access and have access) and sex of the child (male and female) [14].

### Data analyses

First, descriptive analyses were performed using frequency and percentage distributions to examine the characteristics of participants and prevalence of health care seeking behaviour among mothers for children with childhood illnesses. Differences in prevalence were examined using chi-square test. Furthermore, to assess multicollinearity, correlation test was performed among independent variables. The findings showed that the assumptions of multicollinearity were not violated. The tolerance value was greater than 0.10 [42]. Non-response and missing data were excluded to arrive at the weighted sample size. Second, a binary logistic regression model was fitted to examine the relationship between explanatory variables and health care seeking behaviour of mothers. To adjust for sampling variability, DHSs incorporate two-stage cluster sampling [33, 38, 39, 43] and sampling weight was applied to account for the complex survey design including weight, cluster, and strata. Stata version 14 (Stata Corp, College Station, Texas, USA) was used to estimate the prevalence of health seeking behaviour of mothers and odds ratios with 95% confidence intervals (95% CI).

## Results

### Characteristics of the sample population

The descriptive characteristics of respondents are shown in Table 1. The analysis involved a weighted sample of 98,590 under-five children in sub-Saharan African countries, with a fairly even gender distribution (male: 50.7% vs. female: 49.3%). Approximately 54% of children under-5 years of age with acute childhood illnesses did not utilize any health care service. Almost half of mothers were between 25 and 34 years (48.1%). More than one-third of the mothers had primary education (37.6%) and 34.7% had no formal education. A greater proportion of mothers reported they were ever married (93.7%), and 40.0% had no access to media. About 71.1% were living in rural areas, and 39.2% indicated that distance to a health care facility was a problem.

**Table 1:** Prevalence of health care seeking behaviour for children with childhood illnesses

Countries	Seek treatment for sick child	
	Female	Male
Angola	47.8	52.3
Benin	49.2	50.8
Burundi	47.9	52.1
Cameroon	44.7	55.3
Chad	45.6	54.4
Congo DR	49.8	50.2
Ethiopia	48.7	51.3
Gambia	45.6	54.4
Ghana	47.0	53.0
Guinea	46.7	53.3
Lesotho	53.3	46.7
Liberia	45.4	54.6
Malawi	48.8	51.2
Mali	45.1	54.9
Namibia	48.7	51.3
Nigeria	49.2	50.8
Rwanda	49.3	50.7
Sierra Leone	50.2	49.8
South Africa	45.2	54.8
Tanzania	46.1	53.9
Togo	49.9	50.1
Uganda	48.6	51.4
Zambia	49.0	51.0
Zimbabwe	52.1	47.9

### Prevalence of health care seeking behaviour for children with childhood illnesses

Table 1 presents the prevalence of health care seeking behaviour for a sick child in different sub-Saharan African countries. The gender differences in seeking health care were not large. There were preferences for male child care in all countries except Lesotho, Sierra Leone and Zimbabwe. Table 2 also presents the prevalence of health care seeking behaviour of mothers for sick children by socio-demographic characteristics. The prevalence of health care seeking behaviour of mothers for sick children was higher for male children (46.1%), among respondents with formal education (65.3% had at least primary education), women aged 15–24 (46.6%), richer household category (45.8%), never married (47.9%), working (46.5%), exposed to media (47.3%) and those who had no problem (experience no difficulty) accessing the health care services (46.7%). The highest prevalence of health care seeking behaviour among mothers for children with acute childhood illnesses was found in Sierra Leone (65.1%) and the lowest in Cameroon (22.1%) (see Additional file 1).

**Table 2:** Socio-demographic profile and prevalence of health care seeking behaviour for children with childhood illnesses

Characteristics	*N* (98,590)	%	Seek treatment for sick child (*n* = 44,627) 45.3%	*P* value
Seek treatment for sick child				
No	53,963	54.7		
Yes	44,627	45.3		
Sex of child				
Female	48,569	49.3	44.4%	
Male	50,020	50.7	46.1%	0.000
Maternal education				
No formal education	34,229	34.7	42.0%	
Primary	37,023	37.6	46.5%	
Secondary plus	27,338	27.7	47.8%	0.000
Maternal age				
15–24	29,727	30.2	46.6%	
25–34	47,408	48.1	44.9%	
35–49	21,454	21.8	44.2%	0.000
Marital status				
Never married	6203	6.3	47.9%	
Ever married	92,387	93.7	45.1%	0.000
Wealth status				
Poorest	22,700	23.0	44.4%	
Poorer	21,969	22.3	45.3%	
Middle	19,666	20.0	45.7%	
Richer	18,370	18.6	45.8%	
Richest	15,884	16.1	45.3%	0.010
Maternal occupation				
Not working	31,390	31.8	42.6%	
Working	67,200	68.2	46.5%	0.000
Media access				
No access	39,428	40.0	42.2%	
Have access	59,162	60.0	47.3%	0.000
Place of residence				
Rural	70,111	71.1	45.3%	
Urban	28,478	28.9	45.2%	0.777
Distance to health facility				
Experience no difficulty/not a problem	59,969	60.8	46.7%	
Experience difficulty/a problem	38,621	39.2	43.1%	0.000
Number of living children				
1–2	39,385	40.0	47.5	
3–4	31,937	32.4	44.6	
5+	27,268	27.7	42.9	0.000

### Binary logistic regression

The results of the adjusted odds ratios (aOR) and 95% CI for the relationship between socio-demographic variables and health care seeking behaviour of mothers for childhood illnesses are presented in Table 3. The findings showed that the odds of health care service utilization for childhood illnesses were significantly higher among mothers with male children (OR 1.06; 95% CI 1.03–1.08) compared to mothers with female children. The odds of health care service utilization for childhood illnesses were found to be higher among mothers who had primary education (OR 1.19; 95% CI 1.16–1.23) and higher education (OR 1.18; 95% CI 1.14–1.23) compared to those with no formal education. Mothers who were working (OR 1.15; 95% CI 1.12 to 1.18), exposed to media (OR 1.20; 95% CI 1.17–1.23) were more likely to utilize health care service for childhood illnesses compared to their counterparts who were not working or exposed to the media, respectively. The odds of health care service utilization for childhood illnesses were, however, found to be significantly lower among mothers of 3–4 children (OR 0.90; 95% CI 0.87 to 0.93) and mothers of 5+ children (OR 0.85; 95% CI 0.81 to 0.88) compared to those with 1–2 children. In addition, the odds were lower for women in the richer household category (OR 0.90; 95% CI 0.86 to 0.94) and the richest household category (OR 0.85; 95% CI 0.81 to 0.89) compared to the poorest household category. Women who reported that distance was a problem in accessing health care services had lower odds (OR 0.87; 95% CI 0.85 to 0.89) of health care service utilization for childhood illnesses than those who reported that distance was not a problem in accessing health care services.

**Table 3:** Multivariable associations between socio-demographic factors and health treatment seeking behaviour for childhood illnesses

Variables	Adjusted OR	95% CI	*P* value
Sex of child			
Female	1.00		
Male	1.06	1.03 to 1.08*	< 0.001
Age of mothers			
15–24	1.00		
25–34	1.01	0.97 to 1.04	0.761
35–49	1.03	0.98 to 1.08	0.191
Education attainment of mother			
No education	1.00		
Primary	1.19	1.16 to 1.23*	< 0.001
Higher (secondary and above)	1.18	1.14 to 1.23*	< 0.001
Occupation of mother			
Not working	1.00		
Working	1.15	1.12 to 1.18*	< 0.001
Marital status			
Never married	1.00		
Ever married	1.01	0.96 to 1.07	0.679
Wealth index			
Poorest	1.00		
Poorer	0.98	0.94 to 1.02	0.245
Middle	0.97	0.93 to 1.01	0.143
Richer	0.90	0.86 to 0.94*	< 0.001
Richest	0.84	0.80 to 0.88*	< 0.001
Access to media			
No	1.00		
Yes	1.20	1.17 to 1.23*	< 0.001
Distance to health facility			
Not a problem	1.00		
A problem	0.87	0.85 to 0.90*	< 0.001
Number of living children			
1–2	1.00		
3–4	0.90	0.87 to 0.93*	< 0.001
5+	0.85	0.81 to 0.88*	< 0.001

*p < 0.05

## Discussion

This study investigated the predictors of health care service seeking behaviour among mothers for childhood illnesses in sub-Saharan African countries. Overall, 98,590 children under-5 years of age in sub-Saharan African countries born within 5 years preceding the surveys, and reported incidence of diarrhea and/or cough or fever in the past 2 weeks before the surveys [14] were included in the study. The findings revealed disparities in health care seeking behaviour among mothers for children with acute childhood illnesses in sub-Saharan African countries. Hence, much is needed to be done to enhance health care seeking behaviour among mothers for children with acute childhood illnesses. For instance, the highest prevalence of health care seeking behaviour of mothers for children with acute childhood illnesses was found in Sierra Leone (65.1%), while the lowest was found in Cameroon (22.1%), (see Additional file 1). These findings reinforce the need for concerted efforts to enhance health care seeking behaviour of mothers for children with acute childhood illnesses in the region [18].

Overall, less than half (45%) of under-five children with acute childhood illnesses utilized health care services, consistent with previous findings [1, 34, 37, 44]. Low health care seeking behaviour among mothers has been shown to be a major determinant of childhood morbidity and mortality in sub-Saharan African countries [22, 45], which has been attributed to child, social and maternal factors [1, 13, 33, 34]. For example, there is evidence of an association between mothers’ education and health care seeking behaviour for their children, where women with higher levels of education are more likely to seek health care services for their children [33, 46, 47]. Thus, proper health care seeking behaviour of mothers could prevent and reduce childhood illnesses [10, 36, 37], and   childhood mortality in low-income and middle-income countries [1, 34]. In many sub-Saharan African countries, poor and inadequate medical facilities and poor health seeking behaviour are known risk factors for infant and child mortality [1].

In this study, we found sex of child, number of living children, education, work status, wealth index, exposure to media and distance to a health facility to be predictors of health care seeking behaviour of mothers for children with acute illnesses. We observed higher odds of health care seeking behaviour for male children as compared with female children. This finding corroborates previous findings [48, 49], where culture and traditional beliefs have been implicated for this outcome [50]. Male-child preference by some cultures and traditional expectations of men as breadwinners for families are some of the reasons for the extra-care of male children [50]. Furthermore, we observed that education is an important factor in mothers’ health care seeking behaviour, especially for childhood illnesses [2, 35, 51, 52]. Thus, enhancing women’s educational levels will help advance health care seeking behaviour for childhood illnesses [45, 48, 53].

The higher odds of health care seeking behaviour found among employed mothers [1] may be linked to empowerment [54–56], and women’s ability to decide on certain maternal issues [55] and pay for health care service [55, 56]. Health care seeking behaviour of mothers had been associated with skilled or semi-skilled employment [36], which can also be linked with women empowerment. Low health care seeking behaviour among the "richer and richest" wealth indices contrast other findings from previous study [2, 14, 57, 58]. This study speculates that geographical location, educational attainment of women and level of awareness may explain the differences in findings. Meanwhile, low socio-economic conditions including poverty has also been shown to be a factor influencing the attitude of mothers towards seeking health care services for their children [36]. For instance, children might not get the required medical attention due to the mothers’ inability to pay for health services. Prior studies also noted the importance of access to health care facilities in enhancing mothers health care seeking behaviour, and further reducing childhood illnesses [1, 2, 48, 51].

## Strengths and limitations

The study has both limitations and strengths. One of the strengths of the study is that the data sets are from several sub-Saharan African countries and are nationally representative. This permits generalisation of the results in sub-Saharan African countries, despite the time lag in data collection periods. However, comparison of result from different surveys should be done carefully and with caution due to differences in data collection periods [56]. Furthermore, the sample size is sufficiently large as it increases the validity of the findings. Nonetheless, the study is subject to social desirability and recall bias [50, 59], because it was based on self-reported data [59]. Finally, the surveys are cross-sectional, and they only permit association and not causal relationship [30, 38].

## Conclusion

The study analysed secondary data from 24 sub-Saharan African countries on acute childhood illnesses and health care seeking behaviour of mothers in sub-Saharan Africa. The findings indicate a low prevalence of health care seeking behaviour of mothers for childhood illnesses. Socio-economic factors including maternal education were found to be associated with health care seeking behaviour. Public health policies and programmes that target women's empowerment and development are critical to addressing the issue. This may help increase the prevalence of health care seeking behaviour of mothers for childhood illnesses, and consequently improve child health outcomes in sub-Saharan African countries.

## Supplementary Information

**Additional file 1.** Survey characteristics.

## Data Availability

Data used in this study were obtained from the DHS Program and available at: https://dhsprogram.com/data/available-datasets.cfm.

## Abbreviations

AOR: Adjusted odds ratio
CI: Confidence interval
DHS: Demographic and Health Survey
IMCI: Integrated Management of Childhood Illness
WHO: World Health Organization
