# Supplementary material for: Health care seeking behaviour for children with acute childhood illnesses and its relating factors in sub-Saharan Africa: evidence from 24 countries
Source: Trop Med Health. 2021 Dec 14;49:95. doi: 10.1186/s41182-021-00385-1 (PMC8670049; doi:10.1186/s41182-021-00385-1)
Supplement: Supplementary file 1 — Additional file 1. Survey characteristics. [file 41182_2021_385_MOESM1_ESM.pdf]

**Country**	**Survey year**	**N**	**Seek medical treatment (%)**
Angola	2015-16	3747	43.6
Benin	2017-18	3979	25.4
Burundi	2016-17	7976	53.4
Cameroon	2018	3065	22.1
Chad	2014-15	6679	24.2
Democratic Republic of Congo	2013-14	8390	36.9
Ethiopia	2016	3057	28.8
Gambia	2013	2199	62.7
Ghana	2014	1521	56.6
Guinea	2018	2212	41.6
Lesotho	2014	1083	51.9
Liberia	2013	2795	51.6
Malawi	2015-16	8116	56.3
Mali	2013	2890	27.2
Namibia	2013	1965	55.9
Nigeria	2018	10823	61.8
Rwanda	2014-15	2831	45.4
Sierra Leone	2013	3738	65.1
South Africa	2016	1197	44.5
Tanzania	2015-16	2949	28.7
Togo	2013-14	2599	32.4
Uganda	2016	8485	54.1
Zambia	2013-14	3289	54.0
Zimbabwe	2015	3005	28.3
Total		98590	
